# Tissue metabolomics reveals metabolic dysregulation associated with intimal hyperplasia in arteriovenous fistula stenosis

**DOI:** 10.3389/fphys.2025.1638179

**Published:** 2025-08-18

**Authors:** Ming Zhao, Qixin Wu, Yifei Zhao, Rui Nian, Wanjun Li, Hongzhao Lu

**Affiliations:** ^1^ Department of Nephrology, 3201 Hospital, Hanzhong, Shaanxi, China; ^2^ School of Biological Science and Engineering, Shaanxi University of Technology, Hanzhong, Shaanxi, China; ^3^ School of Medicine, Nanchang University, Nanchang, Jiangxi, China

**Keywords:** metabolomics, end-stage renal disease, arteriovenous fistula stenosis, biomarkers, intimal hyperplasia

## Abstract

**Objective:**

This study performed untargeted LC-MS metabolomics on venous tissues from maintenance hemodialysis patients undergoing arteriovenous fistula (AVF) reconstruction surgery.

**Methods:**

A total of six stenotic and six non-stenotic AVF tissues were analyzed. Paired samples were collected from stenotic AVF segments and non-stenotic regions (control group). Histological analysis revealed significant intimal hyperplasia in stenotic tissues (687.90 ± 149.00 μm vs. 286.70 ± 95.18 μm, P < 0.0001 by HE staining) and excessive collagen deposition (Masson staining).

**Results:**

Metabolomic profiling identified 802 metabolites, with 356 differentially expressed (VIP > 1, P < 0.05), predominantly lipids/lipid-like molecules. KEGG enrichment highlighted five dysregulated pathways (P < 0.01): Arginine/proline metabolism; Glycerophospholipid metabolism; ABC transporters; Choline metabolism in cancer; Retrograde endocannabinoid signaling. Six metabolites showed perfect diagnostic potential (AUC = 1.0): niacin, free carnitine, 3-hydroxynonyl-5,7-dienoylcarnitine, 3-methylheptanediylcarnitine, dec-7-enoylcarnitine, and γ-aminobutyric acid. Significant metabolite-clinical correlations included: Choline positively correlating with serum phosphorus (r = 0.62, P = 0.008); Carnitine associating with hemoglobin levels (r = 0.58, P = 0.012).

**Conclusion:**

This tissue-based metabolomics study defines specific metabolic disturbances driving AVF stenosis, proposing mechanistic insights and candidate biomarkers.

## 1 Introduction

Arteriovenous fistula (AVF) is the preferred vascular access for maintenance hemodialysis due to its superior long-term patency and lower complication. However, its clinical efficacy is severely compromised by a high incidence of stenosis, primarily driven by pathological intimal hyperplasia (Sidawy et al., 2002). Despite advancements in imaging and surgical techniques, the prevention and early detection of AVF dysfunction remain major challenges in end-stage renal disease (ESRD) management.

Intimal hyperplasia, characterized by vascular smooth muscle cell proliferation and extracellular matrix deposition, is a key contributor to AVF stenosis and failure. This process shares multiple features with other vascular proliferative diseases, including atherosclerosis and restenosis after angioplasty, suggesting common underlying mechanisms involving vascular remodeling and metabolic dysregulation (Bozzetto et al., 2023; Yan et al., 2024). However, the molecular basis of these processes in the context of AVF remains incompletely understood.

Metabolomics, as an emerging systems biology approach, enables comprehensive profiling of small-molecule metabolites and provides new opportunities to investigate the biochemical alterations underlying complex vascular pathologies. Previous metabolomics studies have primarily relied on blood samples, which are susceptible to systemic confounders and may not accurately reflect local tissue-level metabolic changes (Schlosser et al., 2024; Meyers, 2022). In contrast, direct metabolomic profiling of stenotic AVF tissue offers the potential to uncover more specific metabolic signatures associated with intimal hyperplasia (Anwar et al., 2015; Klusman et al., 2023).

In this study, we performed untargeted metabolomics analysis using high-resolution liquid chromatography–mass spectrometry (LC-MS) on AVF venous tissue from patients with and without stenosis. We aimed to identify differentially expressed metabolites and disrupted metabolic pathways that may contribute to AVF failure, and to explore their potential as early biomarkers for clinical application. Our findings provide novel insights into the metabolic underpinnings of AVF stenosis and offer a new perspective for personalized intervention in ESRD vascular access management.

## 2 Materials and methods

### 2.1 Sample collection

AVF venous tissue samples were obtained from patients undergoing maintenance hemodialysis at the 3201 Hospital (Hanzhong, China) in 2024. All enrolled patients underwent AVF reconstruction surgery, during which stenotic and non-stenotic vein segments were collected. The study protocol was approved by the Institutional Review Board of the 3201 Hospital (Approval Number: Yuan Lun Li Shen [2023] No. 033), and written informed consent was obtained from all participants.

### 2.2 Study subjects

Control cohort: The vascular tissue collected prior to autogenous arteriovenous fistula (AVF) hyperplasia (Control group).

Hyperplasia cohort: Vascular tissues obtained from sites of arteriovenous fistula hyperplasia (AVF group).

Fixation and Preservation: Vascular tissues were excised aseptically from predetermined anatomical sites, immediately frozen in liquid nitrogen, and stored at −80°C.

Clinical information: patient demographics and laboratory indicators, was retrieved from the hospital’s electronic medical record system during the time of AVF tissue sampling.

### 2.3 Preparation of quality control (QC) samples

Equal volumes of metabolites from all samples were pooled to generate QC samples. One QC sample was inserted after every 5 to 15 experimental samples during analysis.

### 2.4 Instrumentation and reagents

Untargeted metabolomic profiling was performed using an UHPLC-Orbitrap Exploris 240 mass spectrometer (Thermo Fisher Scientific, United States) coupled with an Ultimate 3000 UHPLC system. Metabolites were extracted by protein precipitation with cold methanol, followed by centrifugation and supernatant collection. All solvents used were LC-MS grade (methanol, acetonitrile, formic acid, water, and isopropanol from Fisher Scientific, United States). Quality control (QC) samples were prepared by pooling equal volumes of each sample and were injected at regular intervals throughout the analytical sequence to assess system stability and reproducibility. Data acquisition was conducted in both positive and negative ionization modes.

### 2.5 Tissue staining

Vascular tissue specimens were fixed in 4% paraformaldehyde, embedded in paraffin, and sectioned into serial slices of 4 µm thickness. Hematoxylin-eosin (HE) staining (Wei et al., 2024), Masson’s trichrome staining (Hu et al., 2024).

### 2.6 Data processing and metabolite identification

Raw LC-MS data were processed using Progenesis QI software for peak alignment, retention time correction, and normalization. Metabolite identification was based on accurate mass, MS/MS fragmentation patterns, and comparison with public databases including HMDB (http://www.hmdb.ca/) and METLIN (https://metlin.scripps.edu/), as well as the self-constructed Majorbio database.

### 2.7 Statistical analysis

The resulting data matrix was uploaded to the Majorbio Cloud Platform (www.majorbio.com) for multivariate statistical analysis. Principal component analysis (PCA) and orthogonal partial least squares-discriminant analysis (OPLS-DA) were employed to visualize group separation. Variable importance in projection (VIP) values >1 and p-values <0.05 were used to select significantly altered metabolites. Kyoto Encyclopedia of Genes and Genomes (KEGG) pathway enrichment analysis was performed to identify significantly affected metabolic pathways.

## 3 Results

### 3.1 Observation of tissue sections in AVF and control groups

**FIGURE 1:**
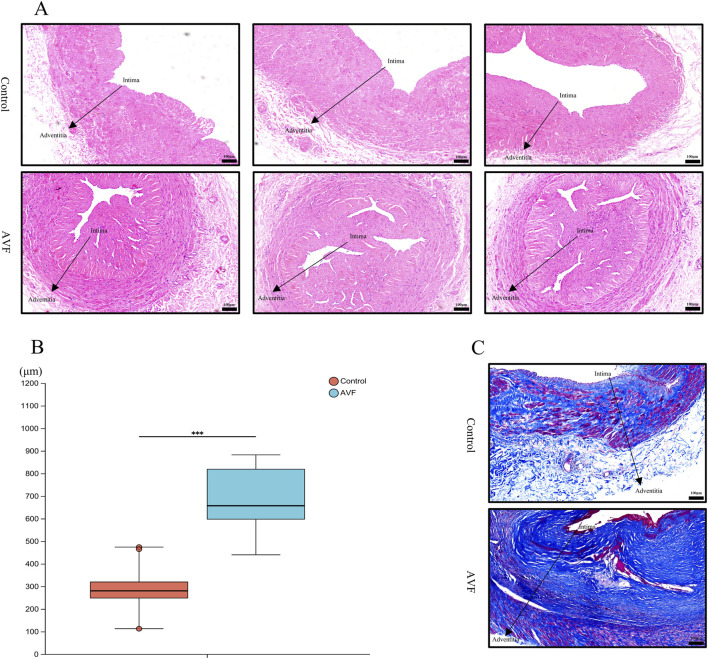
Comparison of histology and fibrosis degree between the Control group and the AVF group. **(A)**, Representative HE-stained images from three different specimens in each group, showing consistent histological features. All images were taken at the same magnification (Scale bar = 100 μm); **(B)**, Quantification of intimal area proportion in AVF and control groups (n = 6 per group). The intimal area was significantly increased in the AVF group compared with the control group. ***P < 0.001, Welch’s t-test.; **(C)**, Representative Masson-stained section of vein (Scale bar = 100 μm).

In the HE-stained venous sections, disordered cellular arrangement, evident proliferation and accumulation, significant intimal thickening, a blurred boundary between the intima and media, and an irregular intimal surface were observed in the AVF group compared to the control group, intuitively indicating intimal hyperplasia (Figure 1A). Quantitative analysis of the HE-stained venous sections revealed that vascular thickness was significantly increased in the AVF group compared to the control group [(286.7 ± 95.2) μm vs. (687.9 ± 149.0) μm, P < 0.0001], indicating a statistically significant difference (Figure 1B).

Collagen deposition was assessed using Masson staining. Compared with the control group, abundant blue-stained collagen fibers were observed in the AVF group, particularly around the vessels, displaying pronounced proliferation and a dense, disordered distribution (Figure 1C). These findings indicate that excessive deposition of extracellular matrix collagen fibers during intimal hyperplasia leads to irregular luminal morphology and deformation, subsequently affecting blood flow and vascular function.

The results of HE and Masson staining demonstrated significant intergroup differences in cellular morphology and arrangement, intimal thickness, and both the distribution and content of collagen fibers.

### 3.2 Metabolic characteristics analysis

**FIGURE 2:**
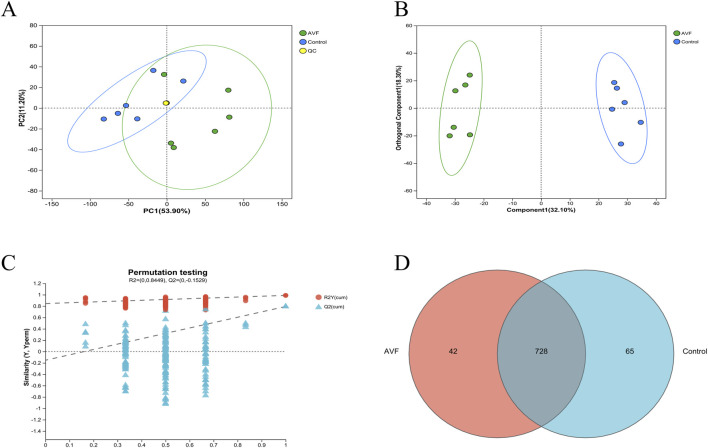
Multivariate Statistical Analysis of Control Group and AVF Group. **(A)**, Principal Component Analysis (PCA) plot of the Control group, AVF (Arteriovenous Fistula) group, and QC samples; **(B)**, Orthogonal Partial Least Squares-Discriminant Analysis (OPLS-DA) plot of the Control group and AVF group (VIP value >1 indicates a metabolite’s significant contribution to group separation in the OPLS-DA model.); **(C)**, Permutation test plot of the OPLS-DA model (R^2^Y represents the goodness of fit of the model, while Q^2^ reflects its predictive performance.); **(D)**, Venn diagram of differential metabolites between the Control group and AVF group.

Untargeted metabolomics was employed to analyze the metabolic profiles of the AVF and control groups, resulting in the detection of 802 metabolites across both groups. To assess potential differences between the two groups, PCA and OPLS-DA were performed on the GC-MS results. As illustrated in Figure 2A, the first principal component (PC1) and the second principal component (PC2) contributed 53.90% and 11.20% of the variance, respectively, with a combined contribution of 65.10%. A clear separation between the two groups was observed in the OPLS-DA score plot (Figure 2B), indicating substantial alterations in their metabolic profiles. To explore metabolite alterations associated with intimal hyperplasia in autologous arteriovenous fistula, the OPLS-DA model was utilized to validate global metabolic differences between the groups. As shown in Figure 2C, the OPLS-DA model exhibited a strong fit and high predictive ability, with R^2^Y exceeding Q^2^ and the Q^2^ regression line intercepting the Y-axis at 0 and -0.1529. These results further support significant alterations in tissue metabolic characteristics during intimal hyperplasia. A Venn diagram analysis was conducted to examine overlapping differential metabolites, revealing 728 commonly expressed metabolites that may serve as potential biomarkers (Figure 2D).

### 3.3 Identification of differential metabolites

**FIGURE 3:**
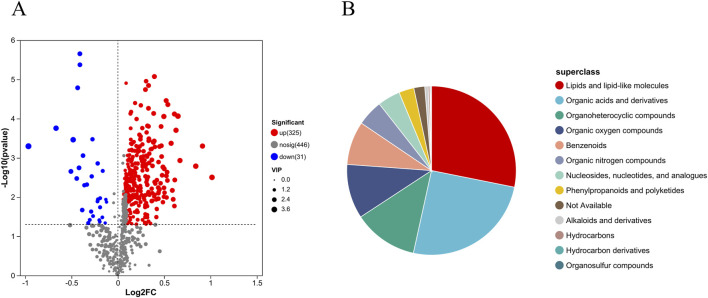
Differential metabolite expression between groups **(A)**, Volcano plot of metabolite differences between groups (Differential metabolites were selected based on P < 0.05, VIP >1, and fold change ≥1.); **(B)**, HMDB compound classification pie chart.

To characterize the tissue metabolic profile of AVF patients and identify high-confidence metabolites associated with venous intimal hyperplasia, differential metabolites with VIP >1, P < 0.05, and fold change ≥1 (based on OPLS-DA) were selected from the 728 shared metabolites and visualized using volcano plots. Compared to the control group, 325 metabolites were upregulated and 31 were downregulated in the AVF group (Figure 3A). Based on the HMDB database, 356 metabolites were further classified and visualized. The results presented in Figure 3B demonstrate that the majority of differentially expressed metabolites were categorized as lipids and lipid-like molecules.

### 3.4 Metabolite correlation and pathway enrichment

**FIGURE 4:**
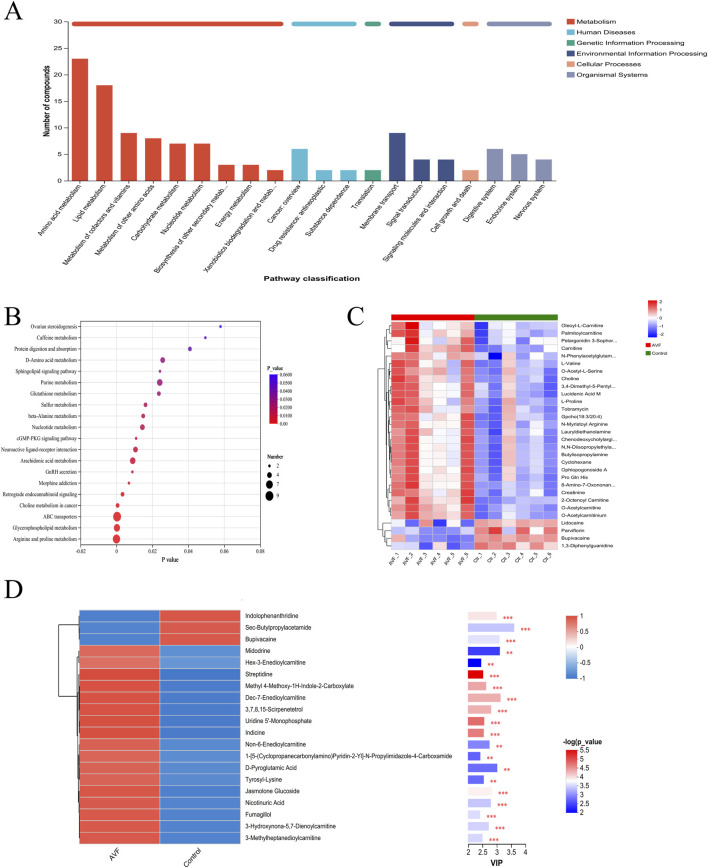
Integrated Pathway and Functional Analysis of Between-Group Differential Metabolites **(A)**, KEGG statistical chart (Enrichment results were based on automated KEGG annotations); **(B)**, KEGG pathway enrichment bubble map; **(C)**, Heatmap of Differential Metabolite Clustering; **(D)**, Differential metabolite vip plot between groups (red indicates higher expression in the AVF group, while blue indicates higher expression in the control group. P-values reflect statistical significance, and VIP scores indicate each metabolite’s importance in the multivariate model. The top color bar denotes the compound class).

To explore the associations between metabolites and regulatory pathways, 356 metabolites were mapped to KEGG functional pathways to investigate their links with relevant phenotypes. The results revealed that lipid and amino acid metabolism were prominently enriched across 20 KEGG-defined pathways (Figure 4A), consistent with the classification trends observed in the HMDB database. To identify the primary KEGG pathways associated with the dysregulated metabolites, 356 differential metabolites were further enriched across 20 metabolic pathways. Based on enrichment factors and P-values, five KEGG pathways were significantly enriched: arginine and proline metabolism, glycerophospholipid metabolism, ABC transporters, choline metabolism in cancer, and retrograde endocannabinoid signaling. Notably, these findings were highly consistent with the metabolite functional enrichment results (Figure 4B).

The top 30 metabolites most strongly associated with venous intimal hyperplasia were selected from the 356 significantly altered metabolites (P < 0.05, VIP >1) for hierarchical clustering and heatmap generation (Figure 4C). According to the HMDB classification, lipid and lipid-related metabolites were found to be upregulated. The subsequently generated VIP plot (Figure 4D) demonstrated that, after the exclusion of drug-related metabolites, five acylcarnitines and niacin remained significantly associated, consistent with the KEGG pathway enrichment results (P < 0.01).

### 3.5 Discovery of candidate biomarkers for venous intimal hyperplasia

**FIGURE 5:**
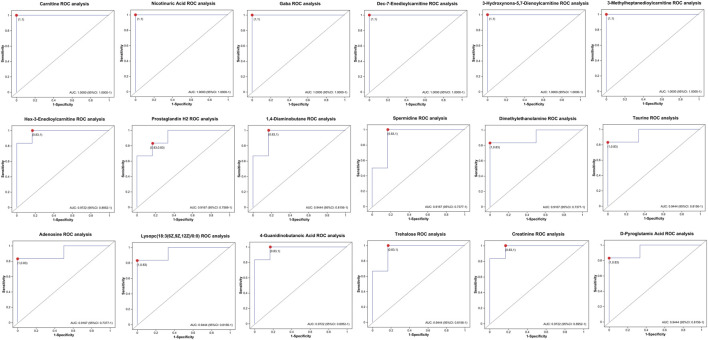
Roc curve analysis of between-group differential metabolites in the hyperplasia versus control Cohorts (AUC ≥0.90).

To further identify potential biomarkers associated with intimal proliferation in the AVF group, receiver operating characteristic (ROC) curve analysis and heatmap visualization were employed. To minimize confounding effects from medications, drug-related metabolites were excluded. Subsequently, metabolites with the top 20 OPLS-DA VIP values and an area under the ROC curve (AUC) ≥0.90 were selected for ROC curve analysis (Figure 5). Among the identified candidates, niacin, free carnitine, three acylcarnitines (3-hydroxynonyl-5,7-dienoylcarnitine, 3-methylheptanediylcarnitine, and Dec-7-enoylcarnitine), as well as γ-aminobutyric acid (GABA), exhibited perfect diagnostic performance with an AUC of 1.0.

### 3.6 Correlation analysis of differential metabolites with clinical indicators

**FIGURE 6:**
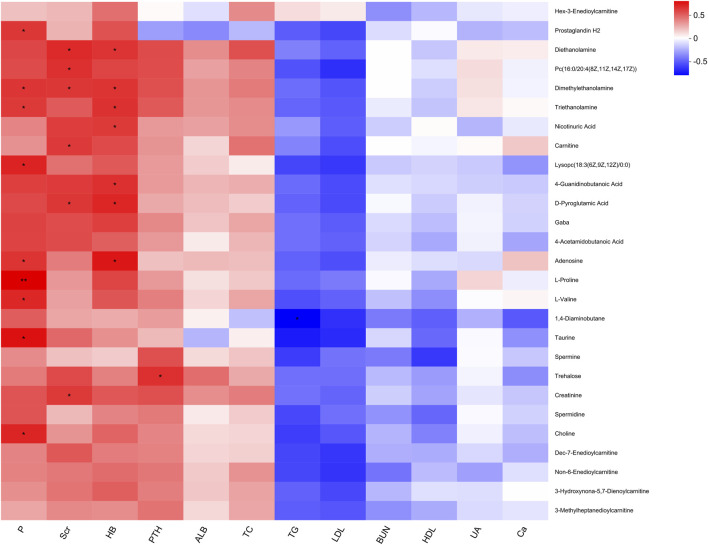
Heatmap of correlation between significant differential metabolites and clinical indicators.

To investigate the potential associations between significantly altered metabolites and clinical indicators, Spearman’s rank correlation analysis was performed, and the results were visualized using a heatmap. As shown in Figure 6, several clinical indicators exhibited significant correlations with specific metabolites in the hyperplasia group (P < 0.05). Phosphorus (P) showed positive correlations with choline, taurine, L-valine, L-proline, adenosine, and lysophosphatidylcholine (18:3 (6Z,9Z,12Z)/0:0). Serum creatinine (Scr) was positively correlated with creatinine, D-pyroglutamic acid, carnitine, and phosphatidylcholine (16:0/20:4 (8Z,11Z,14Z,17Z)). Hemoglobin (HB) exhibited positive associations with adenosine, D-pyroglutamic acid, 4-guanidinobutanoic acid, and nicotinic acid. Parathyroid hormone (PTH) showed a positive correlation with trehalose, while triglycerides (TG) were negatively correlated with 1,4-diaminobutane.

## 4 Discussion

AVF dysfunction, a critical complication in haemodialysis for patients with ESRD, is frequently initiated by intimal hyperplasia and abnormal vascular remodeling, ultimately resulting in access failure. The metabolic profile of AVF has been shown to differ significantly from that of other vasculoproliferative conditions, suggesting that its distinct mechanical adaptation interacts dynamically with the local microenvironment. Metabolic alterations, identified through high-resolution mass spectrometry (HRMS) combined with multivariate statistical analysis, have been implicated in driving pathological vascular remodeling through several mechanisms.

### 4.1 Lipid metabolic remodeling and vascular pathology

Lipid and lipid-like molecules represented the most abundant class of differential metabolites identified between the AVF and control groups. These metabolites play multifaceted roles in vascular remodeling, influencing vascular smooth muscle cell (VSMC) proliferation, intimal dysfunction, and inflammatory responses (Chait and Den Hartigh, 2020). Accumulated fatty acids and dysregulated vascular tone are known to enhance the expression of vascular intimal growth factor (VEGF), thereby promoting pathological hyperplasia. Additionally, lipid mediators exhibit both pro- and anti-inflammatory functions (Robinson et al., 2022). For instance, prostaglandins derived from arachidonic acid contribute to leukocyte recruitment and local inflammation (Gomez et al., 2013), while epoxy fatty acids exert anti-inflammatory effects through suppression of NF-κB and activation of peroxisome proliferator-activated receptors (Hashimoto, 2019). Notably, the lipid metabolic profile in AVF appears to differ from that of other vasculoproliferative conditions. In AVF, lipid alterations are more aligned with atherosclerotic-like mechanisms, including LDL accumulation and macrophage-driven inflammation (Khovidhunkit et al., 2004), whereas in adipose tissue–related vascular expansion, lipid metabolism tends to support vasodilation and triglyceride storage.

### 4.2 Amino acid metabolism in hypoxic vascular remodeling

In addition to lipid dysregulation, KEGG enrichment analysis highlighted significant involvement of amino acid metabolism, particularly pathways involving tryptophan, tyrosine, arginine, and branched-chain amino acids (BCAAs). Tryptophan and tyrosine derivatives are known to influence intimal cell proliferation and vascular homeostasis through mechanisms involving nitric oxide (NO) synthesis, mTORC1 signaling, and oxidative stress (Li et al., 2022; Oberkersch and Santoro, 2019). In the hypoxic and high-flow environment of AVFs, intimal NO synthase activity is suppressed, contributing to impaired NO production and vascular dysfunction (Somarathna et al., 2020). Concurrently, arginase (Arg-I) expression is upregulated, which further limits NO bioavailability and contributes to inflammation modulation (Rath et al., 2014). Moreover, dysregulated BCAA metabolism activates the mTORC1 pathway, promoting inflammation and oxidative stress (Knol et al., 2024). In contrast to AVF, non-AVF vascular lesions such as those associated with obesity often exhibit S-adenosylmethionine–driven epigenetic modifications, including histone H3K36 methylation and macrophage-mediated inflammation (Yu et al., 2019). These observations suggest that amino acid metabolism in AVF is primarily shaped by hypoxia-induced local stress, differing from the systemic metabolic drivers observed in other pathologies.

Given the strong association between amino acid metabolism and vascular remodeling, targeted modulation of arginase activity or restoration of NO signaling may represent promising strategies to alleviate AVF-related intimal dysfunction. Moreover, interventions aimed at rebalancing BCAA metabolism could potentially attenuate mTORC1-driven inflammation and oxidative stress. These avenues warrant further investigation to clarify their therapeutic potential in AVF stenosis.

### 4.3 Integration of key metabolic pathways: KEGG-based mechanistic insights

Five major KEGG pathways—arginine and proline metabolism, glycerophospholipid metabolism, ABC transporters, choline metabolism in cancer, and retrograde endocannabinoid signaling—were significantly enriched among the differential metabolites. Arginine and proline metabolism plays a central role in AVF remodeling. Under hypoxia, Arg-I is activated via the hypoxia-inducible factor (HIF) pathway, redirecting L-arginine from NO synthesis to polyamine production (Yan et al., 2024; Song et al., 2011). This promotes VSMC proliferation, collagen deposition, and intimal dysfunction via increased expression of VCAM-1 and ICAM-1 (Carlström et al., 2024). Proline, a key component of collagen, generates reactive oxygen species (ROS) through its catabolism, activating lymphoid tissue inducer (LTi) cells (Wu et al., 2023). These cells release IL-17 and IL-22 (Cupedo et al., 2008), with IL-17 further promoting neovascularization through STAT3/GIV signaling and VEGF expression (Pan et al., 2015). Together, arginine/proline metabolic reprogramming contributes to collagen remodeling and vascular hyperplasia, and shares partial mechanistic overlap with atherosclerotic lesions.

Glycerophospholipid metabolism, particularly involving phosphatidylcholine (PC) and lysophosphatidylcholine (LPC), also plays a crucial role. Elevated PC inhibits *de novo* lipid synthesis and enhances intimal function via its polyunsaturated fatty acid chains. LPC promotes intimal regeneration by activating fatty acid synthesis but simultaneously exacerbates inflammation through cytokine induction via G protein-coupled receptors (Law et al., 2019). The LPC/PC balance thus modulates vascular homeostasis versus injury.

ABC transporters, especially ABCA1 and ABCG1, mediate cholesterol efflux from vascular cells and reduce inflammation, supporting intimal integrity (Khovidhunkit et al., 2004; Rader and Hovingh, 2014). Their regulatory role in ROS homeostasis and lipid handling suggests a protective effect in vascular remodeling. Similarly, choline metabolism contributes to both PC synthesis and methylation pathways. Choline enhances angiogenesis via α7 nicotinic acetylcholine receptor–mediated HIF-1α and VEGF expression (Jin et al., 2015) and inhibits VSMC proliferation via the M3 receptor (He et al., 2018). However, in inflammatory states, choline uptake through CTL1/ChoKα exacerbates IL-1β–dependent responses, highlighting its dual role in vascular repair and inflammation.

The endocannabinoid system (ECS) represents another enriched pathway. Activation of cannabinoid receptors CB1 and CB2 modulates angiogenesis and inflammation. CB1 promotes helper T cell activity and cytokine secretion, aggravating inflammation, while CB2 suppresses ICAM-1 and VCAM-1 expression, reducing immune cell infiltration (Estrada and Contreras, 2020; Bullock et al., 2023). In AVF, ECS signaling may influence both angiogenic and inflammatory responses during neointima formation.

### 4.4 Biomarker identification and metabolite-function correlation

Among the top 20 differential metabolites, five acylcarnitines and niacin were particularly notable. Acylcarnitines—esters of carnitine and fatty acids—were associated with local oxidative stress and NF-κB–driven inflammation in the AVF anastomotic region (Rutkowsky et al., 2014). In non-AVF lesions, they reflect mitochondrial dysfunction and barrier disruption. Niacin and its metabolite 4PY promote VCAM-1 expression and leukocyte adhesion, facilitating intimal injury (Ferrell et al., 2024). These metabolites showed strong discriminatory power (AUC = 1.0), suggesting their potential as early biomarkers.

GABA, also identified via ROC analysis, exerts both anti-inflammatory and pro-proliferative effects. It reduces inflammation through inhibition of NLRP3 and inflammatory mediator expression, and promotes intimal migration and proliferation (Deng et al., 2024; Wang et al., 2024; Li et al., 2017). Carnitine displays context-dependent roles—enhancing angiogenesis via HIF-1α/VEGF (65), yet inhibiting PDGF-induced VSMC proliferation (Hwang et al., 2020). These findings suggest that metabolic compensation, inflammatory regulation, and intimal recovery may converge through these key metabolites in AVF tissues.

### 4.5 Metabolite–clinical indicator associations

Correlation analyses revealed that choline, carnitine, L-proline, D-pyroglutamic acid, and nicotinic acid were associated with clinical indicators such as phosphorus, creatinine, hemoglobin, and parathyroid hormone. These relationships suggest that intimal hyperplasia may be driven by disruptions in lipid metabolism (e.g., PC and LPC), energy metabolism (e.g., carnitine and adenosine), amino acid metabolism (e.g., BCAAs), and inflammatory signaling (e.g., niacin and taurine). Similar patterns have been observed in metabolic disorders such as NAFLD and obesity (Abdullah et al., 2024; Hawesa, 2024), supporting the involvement of shared pathogenic mechanisms across disease states.

## 5 Conclusion

The metabolic characteristics of vascular hyperplasia in AVF were elucidated through vascular tissue metabolomics, and it was hypothesized that intimal hyperplasia in the AVF group might be promoted by abnormal alterations in multiple metabolic pathways. Alterations in lipid metabolism influence the composition and function of cell membranes. Imbalances in energy metabolism impair normal physiological activity and reduce the proliferative capacity of cells. Dysregulation of amino acid metabolism disrupts protein synthesis and cellular proliferation. Oxidative stress and inflammation initiate a cascade of cellular responses, potentially creating favorable conditions for intimal hyperplasia.

## Data Availability

Datasets are available on request: The raw data supporting the conclusions of this article will be made available by the authors, without undue reservation.
